# CentrosomeDB: a new generation of the centrosomal proteins database for *Human* and *Drosophila melanogaster*

**DOI:** 10.1093/nar/gkt1126

**Published:** 2013-11-21

**Authors:** Joao Miguel da Conceiçao Alves-Cruzeiro, Rubén Nogales-Cadenas, Alberto Domingo Pascual-Montano

**Affiliations:** Functional Bioinformatics Group, National Center for Biotechnology-CSIC, Madrid 28049, Spain

## Abstract

We present the second generation of centrosomeDB, available online at http://centrosome.cnb.csic.es, with a significant expansion of 1357 human and drosophila centrosomal genes and their corresponding information. The centrosome of animal cells takes part in important biological processes such as the organization of the interphase microtubule cytoskeleton and the assembly of the mitotic spindle. The active research done during the past decades has produced lots of data related to centrosomal proteins. Unfortunately, the accumulated data are dispersed among diverse and heterogeneous sources of information. We believe that the availability of a repository collecting curated evidences of centrosomal proteins would constitute a key resource for the scientific community. This was our first motivation to introduce CentrosomeDB in NAR database issue in 2009, collecting a set of human centrosomal proteins that were reported in the literature and other sources. The intensive use of this resource during these years has encouraged us to present this new expanded version. Using our database, the researcher is offered the possibility to study the evolution, function and structure of the centrosome. We have compiled information from many sources, including Gene Ontology, disease-association, single nucleotide polymorphisms and associated gene expression experiments. Special interest has been paid to protein–protein interaction.

## INTRODUCTION

The centrosome in animal cells is a cytoplasmic organelle located near the nucleus, comprised of two cylinders formed by nine microtubule triplets with a highly conserved radial symmetry—the centrioles. The centrioles are embedded in an electron-dense protein matrix, known as the pericentriolar material (PCM), which is basically a meshwork of proteins that nucleates and anchors microtubules and visitor proteins (1). The centriole pair exhibits structural asymmetry, containing one old, mature mother centriole and a young, immature daughter centriole, ∼20% smaller (2). During part of the cell cycle (G1 phase), each cell normally contains only one centrosome. Although, like the DNA, the centrosomes duplicate during the S-phase, in which one daughter centriole forms perpendicularly to each mother centriole. This process results in two centrosomes (each carrying a mother and daughter centriole) connected by a proteinaceous linker (1). This linker will dissolve at the G2/M transition, forming two separate centrosomes (centrosome separation) that can migrate to the poles of the cell and assemble the mitotic spindle, one of its most important functions. Other functions of this organelle in the biology of the cell are related to the organization of the cytoskeleton, the regulation of the cell-cycle and protein regulation processes. Perturbations in the centrosome cycle can have catastrophic consequences, such as centrosome amplification and chromosome instability leading to a variety of human diseases, like ciliopathies and diseases of brain development, or cancer. In fact, although a causal association between centrosome amplification and human cancer development has not yet been firmly established, this condition is frequently implicated as the major mechanism underlying the generation of multipolar mitoses and aneuploidy, and is very often detected in a broad range of tumors, both solid and hematological (3). Moreover, several oncogenic and tumor suppressor proteins localize to the centrosomes, and their deregulation may cause centrosome abnormalities. This collection of emerging data demonstrating the detection of centrosome defects in several preneoplasia has highlighted the centrosome as a novel candidate target for cancer treatment, leading to the growing interest in centrosome biology research that we have witnessed in the last few years (4).

Due to the potential source of new target proteins for further study and characterization, several approaches have tried to identify new centrosomal components, resulting in a whole proteomic characterization of the centrosome. For example, Andersen *et al.* (5) identified 108 centrosomal proteins through a proteomic analysis of the human interphase centrosome; the works of Dobelleare *et al.* (6) identified 32 centrosomal proteins through genome-wide RNA interference, and Muller *et al.* (7) also identified 251 proteins involved in the mitotic Drosophila centrosome. Considering these continuous advances in the characterization of the centrosomal proteome, we sensed the urging need for an updated repository of the results of these and other works, compelling us to present the second version of CentrosomeDB. This new version of the centrosomal database compiles and analyzes the information of likely centrosomal genes of Human and Drosophila organisms from disperse sources of information. In comparison with the first edition of the database, that contained 470 human centrosomal genes (8), CentrosomeDB now owns 1053 centrosomal genes for the *Homo sapiens* and 304 for *Drosophila melanogaster* centrosomal genes, along with some upgrades in the graphical interface and a focus on protein–protein interactions (PPIs).

To the best of our knowledge, there is only one similar database, MiCroKit, that was last updated in July 2009, collecting proteins identified to be localized on kinetochore, centrosome and/or midbody from several species (9). Besides the larger set of genes possessed by CentrosomeDB, we also provide more information on each gene and pay special attention to its graphical representation, resulting in two very distinct databases in the way of treating and analyzing the information. The aim of CentrosomeDB is to significantly improve an important tool for every researcher that works with the centrosome, compiling information from a very broad spectrum of sources of information in one single database, with an easy-to-use but powerful graphical interface.

## NEW FEATURES

### Definition of the set of centrosomal proteins

The new version of centrosomeDB integrates a total of 1357 centrosomal genes, which represents an increase of >190% in comparison with the previous version. In the following descriptions we refer to genes and proteins indistinctly. This is because our database was created in a gene-centric manner. From those 1357, there are 304 centrosomal genes from *D. melanogaster*, a model organism for numerous studies of the centrosome (6,7,10).

These genes were obtained from a vast set of sources of information, from the manual curation of the literature, passing through all the public databases that have proteins or genes annotated as centrosomal and all the way to the orthology relationships with the mouse (*Mus musculus*) centrosomal genes. For each entry in CentrosomeDB there is a three-level ranking scale, representing the strength of the supportive evidence.

### Human centrosomal genes

A total of 147 genes were obtained from the manual curation of scientific publications. To search the literature, we used the following keywords in Pubmed: ‘(centrosome) AND (located OR localiz*)’, selecting ‘human’ as species. We only searched for articles published from our last curated update (01/01/2009), retrieving a total of 320 scientific papers. These articles were manually screened for references to proteins or genes considered or experimentally determined as centrosomal, and were added to the database with the strongest level of confidence. Up to 120 genes were annotated from the Human Protein Reference Database (HPRD) (11) and 469 from the Human Protein Atlas (HPA) (12). These two databases are very complete and valuable, with a lot of genes annotated as centrosomal based on scientific literature and experimental procedures, respectively. The genes extracted from these two databases were compiled in CentrosomeDB with an associated medium level of confidence. A total of 311 genes were collected from gene ontology (GO) (13), using ‘Centrosome’ and ‘Spindle pole body’ as cellular component terms. Gene ontology Biological Processes related to the centrosome, such as ‘centrosome cycle’, ‘centrosome duplication’, ‘centrosome separation’, ‘centrosome localization’ and ‘centrosome organization’ were also considered as evidences of centrosomal localization. In addition, we used the same GO extraction strategy from the *M. musculus* organism, resulting in a list of 222 new centrosomal genes, from which their human orthologs were retrieved and added to our database. The genes supported by GO were assigned the lowest level of confidence for the supportive evidence.

Finally, we used part of the MiCroKit set of genes and included it in CentrosomeDB, with a medium supportive evidence. The MiCroKit database was last updated in 2009 and contains a total of 1489 genes from which 348 are localized to the human centrosome. As with the previous version, we decided to combine both sets of genes, taking into account the small overlap between CentrosomeDB and the MiCroKit—only 119 genes.

After analyzing the evidence codes of supportive centrosomal localization, and the complete list of genes of CentrosomeDB, we observed that 729 of those genes (∼70%) are supported by only one evidence, 145 are supported by two different types of evidences, up to 133 have three evidences, 31 by four evidences and 15 genes are supported by five different supportive evidences. In total, the most frequent sources are the HPA (469), the MiCroKit (348) and the GO database (311). As for the quality of the evidences, we observe that 147 genes have the strongest supportive evidence confidence, 789 genes have the medium and 117 genes have the lowest one (Table 1).

**Table 1.** Summarized table of the number and quality of the supportive evidences of centrosomal localization of the genes of CentrosomeDB

	Human	Fly
Number of genes with the strongest level of confidence	147	229
Number of genes with a medium level of confidence	789	74
Number of genes with the lowest level of confidence	117	0
Number of genes supported by 1 evidence	729	268
Number of genes supported by 2 evidences	145	30
Number of genes supported by 3 evidences	133	6

### *Drosophila melanogaster* centrosomal genes

We decided to upgrade our database and extend its usability for a larger spectrum of researchers by compiling the set of known centrosomal genes of the model organism *D. melanogaster*. To obtain the centrosomal genes, a number of various sources and strategies were followed, including the curation of a large set of scientific bibliography, the browsing of MiCrokit, and the biological database Flybase (14) a huge repository of genetic and molecular data of the family Drosophilidae.

The query ‘(centrosome) AND (located OR localiz*) AND [Drosophila]’ was used in Pubmed, resulting in the curation of 200 articles. Any reference to proteins being localized in the centrosome was used as evidence to the annotation of those genes in our database, with the strongest level of confidence. In total, we included 230 new genes in CentrosomeDB from the curation of the scientific literature, with special relevance to the works of Muller *et al.* (7) (177 new centrosomal genes) and Habermann *et al.* (15) (24 new centrosomal genes). Up to 61 genes that were annotated as centrosomal were retrieved from Flybase and 55 from MiCroKit. These genes were added to CentrosomeDB with a medium level of confidence.

### IMPLEMENTATIONS

CentrosomeDB is a database freely accessible from its website. The site runs over a Ruby on Rails platform (http://rubyonrails.org/) connected to a MySQL server (http://www.mysql.com) that runs in the same computational resource, providing the required information in reduced time. The new network visualization has been implemented by using the Sigmajs javascript library (http://sigmajs.org/).

### Information retrieval

As in the previous version of CentrosomeDB, we used different sources of information to retrieve the data included in the database. The Ensembl system (16), accessed through the R biomaRt package (17), is the main backbone of this work. Given a gene, biomaRt retrieves its description and different synonyms, its isoforms and PDB (18) identifiers, nucleotide and amino acids sequences, its orthologous genes in other organisms, functional information like the associated GO terms and known SNPs variations. Other information has been retrieved directly from its original source. That is the case of OMIM terms (19) and expression experiments from ArrayExpress (20). Pubmed identifiers and related information have been accessed with the eutils point of access at the NCBI (21) while structural information was obtained from Superfamily domains (22) and Pfam domains (23). Coils software (24) was used to predict coiled structures from the protein sequences.

Finally, regarding with the PPIs included in the new release of the database, there are several sources of information that were taken into account. First, we allow scientists to consult the interactions previously reported in other biomedical articles collected in HPRD (11) and Flybase (14) being this search very strict and accurate. On the other hand, we also allow exploring the interactions network space in a deeper way by extending the functional protein networks with the interactions from String database (25), obtaining a wider range interactome. As a result, given a list of genes, we provide five different networks depending on the category of the interacting genes: centrosomal, cyclin and cyclin dependent kinases [according to Swissprot data (26)], and the golgi apparatus and nuclear membrane (according to GO). Interactions are shown for both HPRD or Flybase and STRING.

## THE CENTROSOMAL DATABASE

### Usage

The new version of CentrosomeDB can be accessed from: http://centrosome.cnb.csic.es/, where one can immediately choose between the Human and Fly database. Combining simplicity with power, the website offers a user-friendly graphic interface where the researcher can query the database with a gene name, a database identifier (Esembl, Uniprot, Entrez, Refseq, iPi, Unigene and standard gene name) and searching for specific words of molecular functions or biological processes (full-text mode). It is also possible to search a given domain in our database, or for a specific species, obtaining a list of all the proteins and corresponding genes that contain that domain and a corresponding phylogenetic analysis.

For each gene in the database, the orthologs in other species were identified and compiled, and can be searched for in the field ‘accessing orthology information’. Search by orthology information is also possible. Our database also offers the possibility to blast a given protein sequence as a search option. Downloading is supported by the entire list of genes on a tabular format, with the corresponding evidences of centrosomal localization.

When searching for a centrosomal gene, a large set of valuable information is provided, which makes CentrosomeDB a powerful tool for the study of this organelle. Besides the general information like the localization and the known synonyms, there is a list with all the known protein isoforms—a good example is BRCA1, which has 30 known isoforms. This is followed by the representation of all the known domains of each protein isoform of the gene. The graphical representation of this domain analysis has a special relevance through all CentrosomeDB. Pfam and Superfamily are the two databases that were used to predict the presence of domains in centrosomal proteins. Along with the 3D structure of the protein and information on the GO, CentrosomeDB users can also find information on the known PPIs. Two levels of interactions have been provided. PPI were given a higher importance in this new version, supporting not only interactions with other centrosomal proteins, but also with every other human and/or *D. melanogaster* proteins. This network is filtered so that it only presents the interactions with protein families or organelles that we found, in the literature, to be related to the centrosome, and that might have interesting interactions with it: ‘cyclins’ (27), ‘Cyclin-dependant kinases (CDKs)’ (28), ‘Golgi apparatus’ (29) and ‘nuclear membrane’ (30). When searching for a PPI, one can be redirected to STRING (25), with the same exact search, since all the interactions network of CentrosomeDB were retrieved from this database. In this way, our tool gives the opportunity to study the human and fly centrosomal proteome, from a general perspective, to find new connections between the centrosome and other organelles, new target proteins for future study and characterization, and to identify new molecular pathways, all in an integrated environment. In addition, one can also find any disease-related properties of the gene—information retrieved from ‘Online Mendelian Inheritance in Man’ database OMIM—and a collection of all the scientific bibliography about our specific gene. Finally, all the orthology relationships are summarized in a graph of phylogenetic pattern, from which one can find in which species a gene is either absent or present.

### User case: studying the centrosomal protein interactome

**Figure 1.**
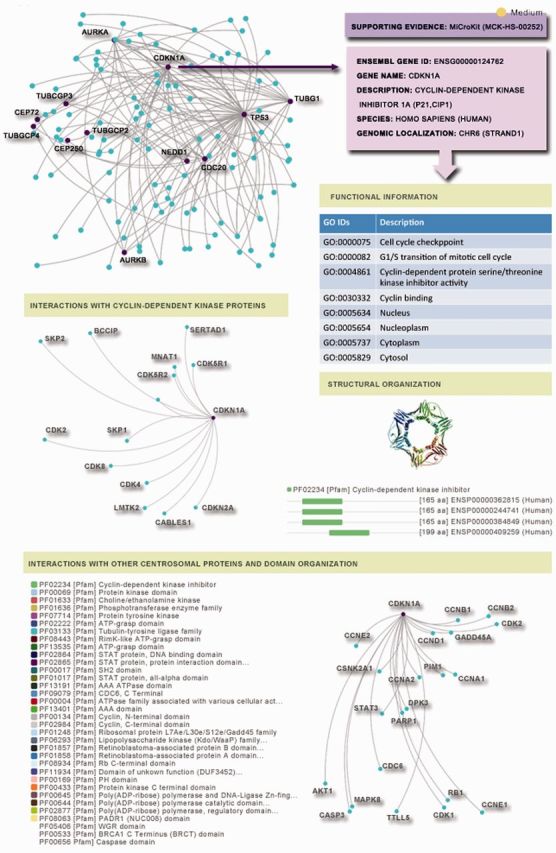
Snapshots of CentrosomeDB results in a typical use case. The Interactome of the centrosomal genes used by Fogeron *et al.* is shown at the top. The CDKN1A protein is selected from the network to visualize its functional information, its interaction with cyclin-dependent kinase proteins, its structural organization as well as its interactions with other centrosomal proteins in the database.

CentrosomeDB navigation is similar to the previous version and it is self-explanatory and intuitive. Therefore to better illustrate its use and potential we focus on one of the new features: the PPIs. The work of Fogeron *et al.* (31)*,* aimed to discover new target molecules that are deregulated in cancer, for their full characterization and study, for example the protein LGALS3BP. With this objective in mind, they expressed 23 centrosomal and cell-cycle proteins in human cells and performed a protein-interaction analysis, creating an interactome against all the known proteins. After this step, they selected 18 out of the original 23 proteins, and created an interactions network against only known centrosomal proteins. We believe that the protein interactions becomes especially important for the study of the centrosome, since the PCM has a meshwork of hundreds of proteins that somehow coordinate with the nuclear regulators of the cell cycle, to assemble the mitotic spindle or to anchor the microtubules that constitute the cytoskeleton. To demonstrate the usage of our database with an example, we first recreated the interactome of centrosomal proteins in our own interactions tool. Then, we selected one gene, and collected all possible information about it, using CentrosomeDB. The resulting interactome can be seen in Figure 1. In total, we managed to create an interactions network, in which 70% of the high-confidence centrosomal interactions of the user case are contained. This percentage refers to direct interactions only. If we consider second level, indirect interactions, we obtain a network that includes 92% of those reported by Fogeron, high confidence and candidate. From the entire interactome we retrieve the four major interactors: TP53, with 189, AURKA, with 176, CDKN1A, with 145 and TUBG1 with 144 interactions. We demonstrate here that interactions studies like the one in this user case can be reproduced with a high fidelity and accuracy, in a considerably easier and more comfortable way. In fact, to be able to observe the interactome of these proteins, *in silico*, a researcher would have to search in any other protein interactions database for each gene, and compile all the data together by selecting only the centrosomal genes. This would be a time consuming and low efficiency method. Our database offers the possibility to search for all the interactions of a list of several genes at the same time, in a centrosomal integrated environment, making it suitable for this kind of studies. Besides the example of this user case, CentrosomeDB offers a resourceful tool to study centrosomal interactions with the other centrosomal proteins and with ‘cyclins’, ‘CDKs’, proteins localized to the golgi apparatus and proteins localized in the nuclear membrane. The interactome of the 18 centrosomal genes with cyclins and CDKs shows a clear peak of the CDKN1A, having 22 interactions with cyclins and 17 with CDKs. Our hope with this kind of analysis is that it may stimulate further research on the relationships between centrosomal components (like CDKN1A) and the proteins that regulate the cell cycle, trying to unravel a little about the signalization pathway that activates the centrosome to assemble the mitotic spindle, during mitosis. Hence, we believe that this feature gives a valuable resource to study the relationships between the centrosomes and some biological processes like the assembly of the mitotic spindle, and also to search for the interactions of a gene with specific molecules, hopefully shedding some light on the function of that gene.

To demonstrate the wide range of action supported by CentrosomeDB we picked a high interacting gene—CDKN1A—and made a full characterization. Figure 1 summarizes all information. The centrosomal localization of CDKN1A is supported by the MiCroKit database, and it is annotated in our database with a medium supportive evidence code. The protein encoded by this gene has four alternative isoforms, according to Pfam, and, as expected, they all have the same unique domain: the CDK inhibitor domain. The list of GO terms associated with CDKN1A is very large, and contains terms such as: ‘G1/S transition of mitotic cell cycle’, ‘cell-cycle checkpoint’, ‘cyclin-dependent protein serine/threonine kinase inhibitor activity’, ‘cyclin binding’—biological process—and ‘nucleus’, ‘nucleoplasm’, ‘cytoplasm’, ‘cytosol’—cellular components.

In addition there are 21 centrosomal proteins that are known to interact with CDKN1A, according to HPRD. CentrosomeDB gives information on the pfam domains of the centrosomal proteins in this interactome. Based on, the most common domains among the interactors of CDKN1A are ‘Protein tyrosine kinase’, ‘Cyclin, N-Terminal domain’, ‘Cyclin, C-Terminal domain’, ‘phosphotransferase enzyme family’ and ‘Protein kinase domain’.

The interactome of CDKN1A with CDKs shows a large network with 15 CDKs, including the CDK2 and the CDK4. It is known that CDKN1A binds to, and inhibits, the activity of the cyclin-CDK2 and cyclin–CDK4 complexes regulating the cell-cycle progression in the G1/S phase (32). All these information is in accordance with the GO terms and the interactome shown by our database, demonstrating in this way the effectiveness and the comfort one can get by using CentrosomeDB.

## DISCUSSION AND FUTURE PERSPECTIVES

The new version of CentrosomeDB now contains two distinct repositories of centrosomal genes, a Fly and a Human database. Besides the huge increment in the number of genes (≥190%), the upgrade focuses also on the PPIs and on the graphical presentation of the information. The large number of genes of this new version comes from an exhaustive curation process that empowers this tool with an extra level of robustness of the information we present. The results presented here are the outcome of several months of manual scrutiny of scientific literature to provide the community with an experimental supportive resource that is difficult to find elsewhere. Besides, each source of the gene information comes with different levels of evidences, which helps in providing a confidence on the data and turns CentrosomeDB in a necessary meeting point of the Centrosome biology.

In the interactions field, when searching for a specific gene, the first version of CentrosomeDB would present a table with the interactions with all the other centrosomal proteins. This approximation was useful to know the centrosomal proteins interactions but it did not allow to explore the relationship between the centrosome and specific biological processes like the regulation of the cell cycle, the assembly of the mitotic spindle or its connection with other organelles or even diseases. This was our motivation for implementing a protein interactions tool in this new version of CentrosomeDB, where users can build an interactome network around this organelle and every other organelle and proteins categories that have been suspected of interacting with the centrosome.

Finally, although CentrosomeDB has compiled a large set of centrosomal genes from other databases, we have directed our efforts towards a different representation of the information, offering different perspectives to study the centrosome domains, orthology information and protein interactions. Also, when researching a gene supported by other known sources, the user can be redirected to the original source.

Looking at the increment in the number of centrosomal proteins with each new study, we believe that, although accurate, our insight of the proteomic constitution of the PCM is still very incomplete. We can only assume that the advancing technologies will permit an increasing number of investigations on the centrosomal proteome and a consequent increase in the number of centrosomal proteins. With this in mind, our objective is to update CentrosomeDB on a regular basis, not only by our efforts, but also with the contribution of the scientific community, from whom we expect an active participation in compiling additional centrosomal genes, or modifying already existing information. A submission form is available, being only necessary to present some sort of supportive evidence on the information to change or add. We are interested in building newer versions of CentrosomeDB, where we could add new cellular components like the cilium/basal body, or even other species that have considerable centrosomal information, like *M. musculus* or the genus Xenopus making CentrosomeDB the best available resource for any scientist studying this organelle.

## FUNDING

The Government of Madrid (CAM) [P2010/BMD-2305]; Spanish Minister of Science and Innovation [BIO2010-17527]; Juan de la Cierva research program (to R.N.C.). Funding for open access charge: Government of Madrid (CAM).

*Conflict of interest statement.* None declared.

## References

[1] Mardin BR, Schiebel E (2012). Breaking the ties that bind: new advances in centrosome biology. J. Cell Biol..

[2] Bornens M (2012). The centrosome in cells and organisms. Science.

[3] Chan JY (2011). A clinical overview of centrosome amplification in human cancers. Int. J. Biol. Sci..

[4] Doxsey S, Zimmerman W, Mikule K (2005). Centrosome control of the cell cycle. Trends Cell Biol..

[5] Andersen JS, Wilkinson CJ, Mayor T (2003). Proteomic characterization of the human centrosome by protein correlation profiling. Nature.

[6] Dobbelaere J, Josué F, Suijkerbuijk S, Baum B, Tapon N, Raff J (2008). A genome-wide RNAi screen to dissect centriole duplication and centrosome maturation in Drosophila. PLoS Biol..

[7] Müller H, Schmidt D, Steinbrink S, Mirgorodskaya E, Lehmann V, Habermann K, Dreher F, Gustavsson N, Kessler T, Lehrach H (2010). Proteomic and functional analysis of the mitotic Drosophila centrosome. EMBO J..

[8] Nogales-Cadenas R, Abascal F, Díez-Pérez J, Carazo JM, Pascual-Montano A (2009). CentrosomeDB: a human centrosomal proteins database. Nucleic Acids Res..

[9] Ren J, Liu Z, Gao X, Jin C, Ye M, Zou H, Wen L, Zhang Z, Xue Y, Yao X (2010). MiCroKit 3.0: an integrated database of midbody, centrosome and kinetochore. Nucleic Acids Res..

[10] Habermann K, Lange BM (2012). New insights into subcomplex assembly and modifications of centrosomal proteins. Cell Division.

[11] Keshava Prasad TS, Goel R, Kandasamy K, Keerthikumar S, Kumar S, Mathivanan S, Telikicherla D, Raju R, Shafreen B, Venugopal A (2009). Human Protein Reference Database–2009 update. Nucleic Acids Res..

[12] Karmali RN, Jones NM, Levine AD (2010). Towards a knowledge-based Human Protein Atlas. Nat. Biotechnol..

[13] The Gene ontology Consortium (2013). Gene Ontology annotations and resources. Nucleic Acids Res..

[14] Marygold SJ, Leyland PC, Seal RL, Goodman JL, Thurmond J, Strelets VB, Wilson RJ (2013). FlyBase: improvements to the bibliography. Nucleic Acids Res..

[15] Habermann K, Mirgorodskaya E, Gobom J, Lehmann V, Müller H, Blümlein K, Deery MJ, Czogiel I, Erdmann C, Ralser M (2012). Functional analysis of centrosomal kinase substrates in Drosophila melanogaster reveals a new function of the nuclear envelope component otefin in cell cycle progression. Mol. Cell. Biol..

[16] Flicek P, Ahmed I, Amode MR, Barrell D, Beal K, Brent S, Carvalho-Silva D, Clapham P, Coates G, Fairley S (2013). Ensembl 2013. Nucleic Acids Res..

[17] Kasprzyk A (2011). BioMart: driving a paradigm change in biological data management. Database J. Biol. Databases Curat..

[18] Rose PW, Beran B, Bi C, Bluhm WF, Dimitropoulos D, Goodsell DS, Prlic A, Quesada M, Quinn GB, Westbrook JD (2011). The RCSB Protein Data Bank: redesigned web site and web services. Nucleic Acids Res..

[19] Amberger J, Bocchini C, Hamosh A (2011). A new face and new challenges for Online Mendelian Inheritance in Man (OMIM®). Hum. Mutat..

[20] Rustici G, Kolesnikov N, Brandizi M, Burdett T, Dylag M, Emam I, Farne A, Hastings E, Ison J, Keays M (2013). ArrayExpress update–trends in database growth and links to data analysis tools. Nucleic Acids Res..

[21] Geer LY, Marchler-Bauer A, Geer RC, Han L, He J, He S, Liu C, Shi W, Bryant SH (2010). The NCBI BioSystems database. Nucleic Acids Res..

[22] De Lima Morais DA, Fang H, Rackham OJL, Wilson D, Pethica R, Chothia C, Gough J (2011). SUPERFAMILY 1.75 including a domain-centric gene ontology method. Nucleic Acids Res..

[23] Punta M, Coggill PC, Eberhardt RY, Mistry J, Tate J, Boursnell C, Pang N, Forslund K, Ceric G, Clements J (2012). The Pfam protein families database. Nucleic Acids Res..

[24] Lupas A, Van Dyke M, Stock J (1991). Predicting coiled coils from protein sequences. Science.

[25] Franceschini A, Szklarczyk D, Frankild S, Kuhn M, Simonovic M, Roth A, Lin J, Minguez P, Bork P, von Mering C (2013). STRING v9.1: protein-protein interaction networks, with increased coverage and integration. Nucleic Acids Res..

[26] UniProt Consortium (2013). Update on activities at the Universal Protein Resource (UniProt) in 2013. Nucleic Acids Res..

[27] Pascreau G, Churchill MEA, Maller JL (2011). Centrosomal localization of cyclins E and A: structural similarities and functional differences. Cell Cycle.

[28] Lacey KR, Jackson PK, Stearns T (1999). Cyclin-dependent kinase control of centrosome duplication. Proc. Natl Acad. Sci. USA.

[29] Hurtado L, Caballero C, Gavilan MP, Cardenas J, Bornens M, Rios RM (2011). Disconnecting the Golgi ribbon from the centrosome prevents directional cell migration and ciliogenesis. J. Cell Biol..

[30] Bolhy S, Bouhlel I, Dultz E, Nayak T, Zuccolo M, Gatti X, Vallee R, Ellenberg J, Doye V (2011). A Nup133-dependent NPC-anchored network tethers centrosomes to the nuclear envelope in prophase. J. Cell Biol..

[31] Fogeron M-L, Müller H, Schade S, Dreher F, Lehmann V, Kühnel A, Scholz A-K, Kashofer K, Zerck A, Fauler B (2013). LGALS3BP regulates centriole biogenesis and centrosome hypertrophy in cancer cells. Nat. Commun..

[32] Broude EV, Swift ME, Vivo C, Chang B-D, Davis BM, Kalurupalle S, Blagosklonny MV, Roninson IB (2007). p21(Waf1/Cip1/Sdi1) mediates retinoblastoma protein degradation. Oncogene.

